# On the Hierarchical Organization of Oscillatory Assemblies: Layered Superimposition and a Global Bioelectric Framework

**DOI:** 10.3389/fnhum.2019.00426

**Published:** 2019-12-04

**Authors:** Ravinder Jerath, Connor Beveridge, Michael Jensen

**Affiliations:** ^1^Charitable Medical Healthcare Foundation, Augusta, GA, United States; ^2^Department of Medical Illustration, Augusta University, Augusta, GA, United States

**Keywords:** oscillation, neural oscillation, metastable, consciousness, layered model, phenomenology, default space, hierarchical model

## Abstract

Bioelectric oscillations occur throughout the nervous system of nearly all animals, revealed to play an important role in various aspects of cognitive activity such as information processing and feature binding. Modern research into this dynamic and intrinsic bioelectric activity of neural cells continues to raise questions regarding their role in consciousness and cognition. In this theoretical article, we assert a novel interpretation of the hierarchical nature of “brain waves” by identifying that the superposition of multiple oscillations varying in frequency corresponds to the superimposing of the contents of consciousness and cognition. In order to describe this isomorphism, we present a layered model of the global functional oscillations of various frequencies which act as a part of a unified metastable continuum described by the Operational Architectonics theory and suggested to be responsible for the emergence of the phenomenal mind. We detail the purposes, functions, and origins of each layer while proposing our main theory that the superimposition of these oscillatory layers mirrors the superimposition of the components of the integrated phenomenal experience as well as of cognition. In contrast to the traditional view that localizations of high and low-frequency activity are spatially distinct, many authors have suggested a hierarchical nature to oscillations. Our theoretical interpretation is founded in four layers which correlate not only in frequency but in evolutionary development. As other authors have done, we explore how these layers correlate to the phenomenology of human experience. Special importance is placed on the most basal layer of slow oscillations in coordinating and grouping all of the other layers. By detailing the isomorphism between the phenomenal and physiologic aspects of how lower frequency layers provide a foundation for higher frequency layers to be organized upon, we provide a further means to elucidate physiological and cognitive mechanisms of mind and for the well-researched outcomes of certain voluntary breathing patterns and meditative practices which modulate the mind and have therapeutic effects for psychiatric and other disorders.

## Introduction

Neural oscillations, or “Brainwaves,” are fluctuations in activity shared among neuronal populations (evident as extracellular voltage fluctuations; Jia and Kohn, 2011) and were first discovered in the late 19th century in animals (Beck, 1890; Coenen et al., 2014). The first electroencephalogram (EEG) was performed by *Berger* in the early 20th century revealing Alpha waves (Berger, 1929) which lead to a volley of research into these waves shortly after. Electromagnetic or EEG synchronization between brain areas indicates functional connectivity between those areas (Ivanitsky et al., 1999). Even though such oscillations are known to be a component of many cognitive functions such as feature binding, neural communication (Fries, 2005), perception (Gray et al., 1989), and information processing (Gupta et al., 2016), it is still debated whether oscillations contribute to these processes or are merely an epiphenomenon (Koepsell et al., 2010). Various frequency bands of oscillations from very slow (<0.1 Hz) to very fast (600 Hz) have been shown to each be correlated to distinct aspects of mental activity (Stookey et al., 1941; Schnitzler and Gross, 2005; Fingelkurts and Fingelkurts, 2010a), and analysis of the EEG can be used to determine one’s level and potentially state of consciousness (Cvetkovic and Cosic, 2011; Fingelkurts et al., 2013).

Neural oscillations provide a powerful means to encode and transfer information in space and time (Cheong and Levchenko, 2010). They are the most efficient mechanism to transfer such information reciprocally between neural assemblies (Buzsáki and Draguhn, 2004). They exist at multiple spatial levels from microscopic to macroscopic which can arise from mechanisms within individual neurons as well as interactions between them (Haken, 1996), all of which are a component of the bioelectric structure we describe. The brainwaves observed on EEG are in fact mesoscopic or macroscopic oscillations (Freeman, 2003). Microscopic oscillations are not as easily detectable. Subthreshold membrane potentials are a major microscopic component of these layers that occur in frequencies observed in an EEG. Just as action potentials and various types and patterns of synaptic connections serve as a means of information representation, computation, and transmission, subthreshold membrane potential oscillations provide a means for individual neurons to be a part of a collective whole (Fingelkurts et al., 2010). Such intrinsic single cell oscillations form the basis for frequencies of mesoscopic activity generated by the summed dendritic activity of many neurons within a neural assembly which can be viewed in an EEG (Başar, 2008). Neuronal assemblies can, in turn, synchronize with other adjacent or distant assemblies to form stronger and more global macroscopic oscillations responsible for the greater neural electromagnetic field (Jirsa and Kelso, 2000). The emergent characteristic of large-scale bioelectric activity provides a metastable bridge to global coherence needed for an integrated experience (Fingelkurts et al., 2010).

Brains are systems that never reach a truly steady-state, constantly changing in dynamic patterns (Freeman, 2007; Fingelkurts et al., 2009). A concept of nonlinear dynamics, metastability in regards to the brain describes the local-global harmony of the brain which may be responsible for the emergence of consciousness; distinct functional modules coupled together *via* neural oscillations while still maintaining their intrinsic, independent behavior (Freeman and Holmes, 2005; Kelso and Tognoli, 2007; Fingelkurts et al., 2013). There is thus competition in brain regions between the tendency to act autonomously and to cooperate macroscopically with other regions (Bressler and Kelso, 2001; Fingelkurts and Fingelkurts, 2001). In this metastable mode of functioning, although there is competition between the stability of either tendency, these local and global tendencies can coexist (Kelso and Engstrøm, 2006). Oscillations may be an optimal metastable mechanism as they provide a low-energy operation for local and distant communication which is lost in action potential signaling in distant axonal connections (Buzsáki and Draguhn, 2004). A relatively large brain with only axonal connections would have severe spatial and metabolic constraints (Knyazev, 2012).

According to the Default Space Theory of Consciousness and other prominent theories on consciousness, consciousness is an emergent phenomenon which arises as the virtual recreation or simulation of the environment and the individual’s relationship to it (Revonsuo, 2006; Fingelkurts et al., 2010; Metzinger, 2013; Jerath et al., 2015a). Metastability, oscillations, and consciousness have been extensively researched as a part of the operational architectonics theory of brain-mind (OA) in an attempt to neurophysiologically explain the integrated experience and mind. The theory we propose here is in line with the OA argument that the virtual structure of conscious experience corresponds to, or is functionally isomorphic to, the structure or architecture of the brain’s electromagnetic field (Fingelkurts and Fingelkurts, 2001; Fingelkurts et al., 2009). Functional isomorphism describes two systems as correlating in a way in which functional relations are always preserved regardless of the physical nature of either system (Shapiro, 2000). For instance, a digital computer can be isomorphic to an analog one if the transitional relations among its physical states mirror those in the analog one (Putnam, 1975). Thus, whatever the phenomenal constitution of consciousness is at a given time, it will be isomorphic to its neural correlate. OA explains in-depth how any phenomenal state/pattern is reflected appropriately to a neurophysiological state/pattern (Fingelkurts et al., 2007). A major assumption and basis of this article is a fundamental of OA, that the phenomenal mind is isomorphic to the globally unified electromagnetic field of the brain which consists of a nested hierarchy of oscillatory activity (Fingelkurts et al., 2010). In this article, we explore a potential implication of an aspect of this architecture that is neglected by most in EEG-based research (Fingelkurts and Fingelkurts, 2010a), that being integrative brain functions arise from the bioelectric architecture of the brain *via* multiple oscillations phasically superimposed upon one another based on frequency (Başar et al., 2004; Başar, 2006). This idea that the true composition of the bioelectric structure consists of a concert of multiple superimposed oscillations is most often neglected as EEG analysis is mostly done by taking different frequency bands in isolation (Fingelkurts and Fingelkurts, 2010a). Thus, the true bioelectric structure of one brain may be vastly different from another while still having identical averaged spectral band results (Fingelkurts and Fingelkurts, 2010a).

OA has described how at the core of the isomorphism between the neurophysiological organization of the brain and the informational organization of the phenomenal mind lies the “operation,” or the bioelectric processes occurring among the (potentially many) neural assemblies of the brain (Fingelkurts and Fingelkurts, 2005). Complex operations of synchronized bioelectric activity among distributed neural assemblies, termed operational modules by OA, allow for metastability as the neural assemblies can do their own tasks while still be synchronized with greater and more abstract operations (Fingelkurts et al., 2009). A potentially infinite nested hierarchy of operational modules, which are at the base level composed of basic operations within neural assemblies, may exist as the simplest modules can become synchronized or abstractly unionized with other modules to form a greater and more abstract module, which can be further unionized with other abstract modules all the way to the most macroscopic level of bioelectric activity proposed to be isomorphic to the integrated experience (Fingelkurts and Fingelkurts, 2005, 2006). While frequency bands are often identified with distinct functions, some authors have discussed how oscillations of different bands may be grouped into intrinsic layers or “wave-sequences” (Steriade, 2006), or at least superimposed upon other spectrally distinct oscillations (Başar et al., 2004). In this theoretical article, in contrast to the traditional view that the localization of higher and lower frequency activities are spatially distinct (Luo et al., 2014), we describe an organization of bioelectric cortical neurodynamics modeled as hierarchical “layers” of oscillatory frameworks differentiated by frequency which are not spatially distinct, but coexist in the same brain regions. The lower layers (low frequency) represent more basic and widespread integrative activity, while the higher layers (high frequency) represent more complex and localized activity. We thus form a further theoretical understanding on the organization of the global bioelectric architecture, referred to as a unified metastable continuum in OA (Fingelkurts et al., 2009), by describing the superimposition of such layers and its role in such a continuum. Although divisions and dynamics between these layers may be complex in reality, in basic modeling of such architecture, each layer we describe can be thought of as an independent functional component of this continuum. The higher layers however are dependent upon the lower ones to be a part of the global architecture as they entrain upon them just as the phenomenal isomorphic counterparts to the higher layers are dependent on the phenomenal isomorphic counterparts of the lower layers.

The fundamental elements of oscillations we see heavily summated (approximately millions of neurons) on an EEG are the ionic current producing membrane potential activities of individual neurons; the dendritic and postsynaptic potentials (Klein and Thorne, 2006). The activity of individual neurons consists of relatively simple electrical activity, and can thus be considered *nonconscious* in contrast to the coordinated conscious and unconscious bioelectric activity of neural assemblies which have a phenomenological ontology (Searle, 1992; Fingelkurts et al., 2010). The phenomenal unity of human experience indicates that there must be some mechanism(s) to unify processes responsible for the many aspects of experience such as the variety of sensory modalities. We agree with the metastable view that the synchronized operations of several neural assemblies that are integrated EEG spatial-temporal patterns allow for the global functional unity (Honey et al., 2007; Werner, 2009) needed for the integrated experience (Fingelkurts and Fingelkurts, 2005). While consciousness has been suggested to be quantized (some states more conscious than others; Oizumi et al., 2014), we focus on the phenomenal qualities and contents of human experience in this article.

**Figure 1:**
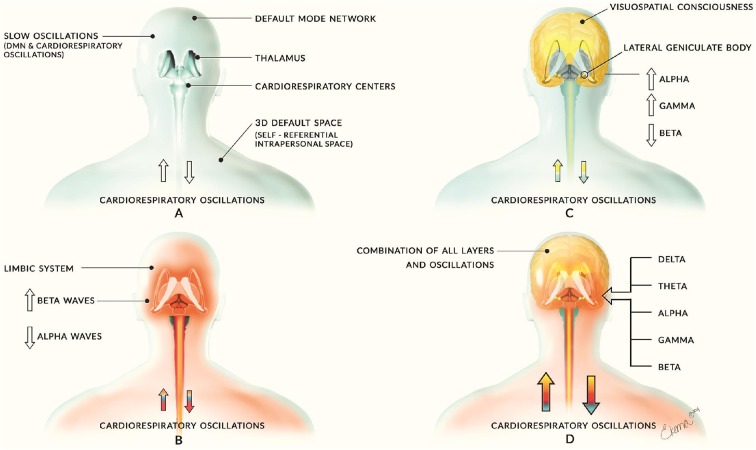
Introductory model of layered activity. This image comes from our initial article on this theory of which we have significantly improved in the updated version. Panel **(A)** illustrates the base layer of slow neural oscillations of the Default Mode Network (DMN) and cardiorespiratory activity. This creates a foundation for all other layers of oscillatory activity and is depicted by the blue coloring. Panel **(B)** shows the second layer of middle-frequency activity largely consisting of limbic activity and is depicted by red coloring. Panel **(C)** reveals the corticothalamic feedback loops involved in cognitive and consciousness processes and is illustrated with yellow coloring. Panel **(D)** combines these layers to form the sum of human neural activity consisting of all neural and physiological oscillations. The multi-colored arrows of each person represent the layers interacting with the cardiorespiratory system with the appropriate color for each layer. Previously published in Jerath and Crawford (2015), permission by CC-BY.

This article may be seen as a further development of our opinion article on this layered model in which we introduced three separate but highly interactive oscillatory frameworks (Jerath and Crawford, 2015). We elucidate an updated model here by detailing each of these layers and exploring their nature in different mental states. In the introductory article, we described a base layer of slow oscillations maintained in part by the Default Mode Network (DMN) and cardiorespiratory activity. The second layer, built upon the first, is a constraining emotional layer powered by the limbic system. The highest layer consists of higher frequency activity among the elements of the corticothalamic network which creates higher cognitive and perceptual components of mind (Figure 1). In this updated version of the model, we have separated the infra-slow oscillations and the Delta oscillations into two layers based on their physiological distinctions and focus on the spectral aspects of these layers rather than anatomical locations. We heavily strengthen our perspective with supporting research and discuss how breathing plays a role in the organization of neural oscillations.

In addition to describing the spectral, layered hierarchical framework relative to a global bioelectric architecture, we further extend the oscillatory framework from the brain to neural and non-neural elements of the body. The relationship between neural oscillations of the brain and activity of the body (largely autonomic) has been explored previously by ourselves and other authors. Coordination and communication between the autonomic bodily system and the brain have been demonstrated in several studies (Walker and Walker, 1983; Basar, 2010). These links reveal the likely existence of bidirectional oscillatory links between organs of the body and the brain which may allow for the maintenance of survival functions such as body temperature (Achimowicz, 1992; Fingelkurts et al., 2011). There is also a link between neural oscillations and the immune system of the body (Saphier et al., 1990; Rosenkranz et al., 2003). In addition, support for the idea that respiration acts as a oscillatory scaffold in the brain is growing (Heck et al., 2016, 2017; Varga and Heck, 2017). Research into this relationship between the brain and body has not explored how this relationship fits into the global architecture. We suggest the body fits (largely respiratory elements) into this architecture and may act as an underlying coordinator of bioelectric neural activity. We also suggest the bioelectric structure of the brain in a sense may be projected to or unified with the sensory receptors of the body.

Although the concept of a hierarchy of brain oscillations across space and time has been previously proposed by notable authors (Freeman, 1987; Lakatos et al., 2005; Knyazev, 2012; Buzsáki et al., 2013; Fingelkurts et al., 2014; Fingelkurts and Fingelkurts, 2017a), we model a hierarchy in a novel way based on frequency by contending that these superimposed spectral layers are isomorphic to superimposed aspects of phenomenal consciousness. Isomorphism among electromagnetic structure and phenomenal structure has been described (Fingelkurts et al., 2009); however, here we describe an isomorphism between the superimposition of electromagnetic “layers” and the superimposition of various components or “layers” of the phenomenal mind. The layers we detail in this article have significantly more functionality, detailed operational processes, and blurred spectral borders, however, the modeling of an oscillatory spectral hierarchy which distinguishes groups of oscillatory networks and how the superimpose in relation to the phenomenal mind may further the understanding of the intrinsic and ubiquitous nature of oscillations in relation to psychology.

## Layers

Current literature supports the assertion on a unified hierarchical nature to neural oscillations which have focused on spatial scales (neurons to cortical columns to functional areas; Fingelkurts et al., 2010, 2013). As a basis of the main assertion of this article, we, however, describe a spectral hierarchy in which higher frequency activity is related to high degrees of consciousness while lower frequencies are associated with the unconscious and subconscious (Roohi-Azizi et al., 2017). Our layered interpretation is unique in its formulation of the layers (although similar ideas have been expressed), the identification of the superpositioning of these layers with the superpositioning of their phenomenal and cognitive counterparts, and the inclusion of aspects of the body into a global oscillatory architecture. The layers of the model may be simply understood metaphorically as the layers of a cake. Each layer is independent, but the layers interact dynamically, and the upper layers depend on the support of the lower layers. In addition, each layer except the base of the “cake” is not horizontally continuous, meaning the cake is not shaped like a cube, but more fanciful like a castle or scene. This means the more localized, higher frequency oscillatory activities of the upper layers in the brain (cake) are not directly connected but are indirectly connected *via* the lower layers (base of cake).

**Figure 2:**
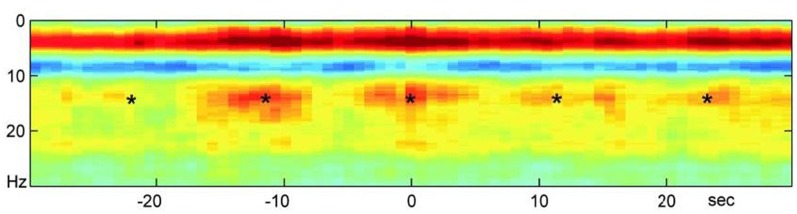
A neocortical power spectrogram and slow oscillatory entrainment. A power spectrogram used to represent neural oscillatory activity maps visually the power of varying frequencies on the spectrum of neural oscillations over time. The spectrogram shown here reveals the normalized average of power spectrograms of a large set of mice. The activity consists of hippocampal ripples over the neocortex during sleep. This spectrogram demonstrates the hierarchical nature of spectral entrainment and superimposition in which slower oscillations modulate the higher ones as well as the propensity for the slow oscillations to synchronize higher frequency activity in different neural areas. The *s represent hippocampal ripples that reveal co-modulation and entrainment of the neocortex and hippocampus by the infra-slow rhythm (0.1 Hz). In addition, this ripple activity is modulated by the Beta activity and both are modulated by the Delta Activity shown in the solid red band indicating its power during sleep. All three layers above the first are in turn biased by the infra-slow rhythm. Clearly, entrainment by slower oscillations exists at multiple levels. Image reproduced from Sirota et al. (2003); permission provided by CC-BY, Copyright 2003 National Academy of Sciences.

Significant literature on oscillations that we explore in this article support the basis of our hypothesis that the lower levels perform global and sustained integration of the many parallel local computations apart of the higher layers (Singer, 2011; Jerath and Beveridge, 2019b). When referring to the “stacking,” or superpositioning, of these layers we refer to phase-phase coupling, although other types of coupling may be involved. A spectrogram illustrating this effect is shown in Figure 2. The domination of higher frequency activity in the EEG in mammals may reflect the evolutionary “advancement” and thus “thickening” of the upper layers of the model. Advancement is quoted because generally, evolution is not considered to proceed in any intended direction (Dawkins, 2004). While the underlying lower layers are not as evident in recordings due to overshadowing by the higher layers, these lower levels may still occasionally dominate local and global oscillatory activity when the higher levels fail due to dysfunction as in epileptic seizures and brain tumors (Karameh and Dahleh, 2000) Higher layer activity may also be restrained to make way for more primitive homeostatic activity such as sleep activity necessary for reconciling experiences with the fundamental biological self (Knyazev, 2012).

There is significant and growing evidence that the global activities of the lower layers and the local activities of the higher layers work in close conjunction in both directions (Knyazev, 2007). The bi-directional dynamic interactions between the layers may include the modulation of high-frequency local activity by large-scale, lower frequency layers as well as the propagation of this local activity to other networks or into global availability (Demiralp et al., 2006) by the lower layers. In the higher layers, the phase-locking (synchronization) characteristics of the waves appear to be related to conscious phenomenology while the power of the waves is related to nonconscious encoding and processing (Melloni et al., 2007). Thus, synchronization may be a key physiological component of consciousness. These layers together form a metastable bioelectric architecture suggested to be isomorphic to the contents of consciousness consisting of a global synchronization of local high-frequency activity (Fingelkurts et al., 2010, 2013) coordinated and mediated by the low-frequency activity and the thalamus (Jerath et al., 2015a; Jerath and Beveridge, 2019b). The layers we describe are not only identified by the frequency of their oscillations but have a phenomenal counterpart as well which is isomorphic to the oscillatory framework. Just as hierarchy exists in the global bioelectric architecture, a hierarchy exists in the complex structure of phenomenal experience (Revonsuo, 2006; Fingelkurts et al., 2013). We share the assertion that at the basis of this hierarchy lays a subconscious, virtual three-dimensional (3D) coordinate matrix in which all phenomenal features are realized and integrated into one experience (Dainton, 2000; Fingelkurts et al., 2013). Phenomenal contents are simultaneously present in experience however these contents are entrained upon or integrated with one another (Fingelkurts et al., 2009). For instance, qualia are superimposed upon objects which are superimposed upon scenes that are all superimposed into the 3D coordinate space. Some objects, such as the body, are superimposed onto a representation quality of the self or internal world while the others are superimposed upon a representation of the external world. Furthermore, abstract concepts such as thoughts and feelings are superimposed into this phenomenal structure in a more abstract sense but still reside within the 3D space and as a component of the representation of the self. Thus, all phenomenal content is coordinated perfectly in time and is unified in space (Fingelkurts et al., 2009). We go on to describe the phenomenal, cognitive, and biological components of the layers and the nature of the isomorphism among the superimposing of the components of their hierarchies.

### Layer One—a Universal and Unifying Network

The base layer of the oscillatory architecture we describe is the most important and fundamental as it provides a widespread foundation for the other layers to build upon and thus is the organizer which groups the layers together. This layer consists of membrane potential and local field potential oscillations which are continuously active and oscillate in the slowest range (<1 Hz). Crucial for neocortical function, the membrane potential of nearly all neocortical neurons undergo 10–20 mV oscillations (Steriade et al., 1993a). While this globally synchronized slow oscillation of Up and Down states is the overriding EEG pattern during non-REM sleep is well studied (Crunelli and Hughes, 2010), little is known about the functional role of this slow oscillation in the awake cortex (Neske, 2016). As other authors have asserted that slow oscillations are overshadowed by fast oscillations (Knyazev, 2012), we suggest this slow oscillation is present in the waking state but dominated in the EEG by faster oscillations which are entrained upon it. In addition, the macroscopic infra-slow bioelectric oscillation (<0.1 Hz) is intrinsic and fundamental to brain functioning and correlates with faster oscillations (Lőrincz et al., 2009).

Slow oscillations do indeed modulate (Sirota and Buzsáki, 2005; Canolty et al., 2006; Schroeder and Lakatos, 2009; Buzsáki and Wang, 2012), group (Vanhatalo et al., 2004; Steriade, 2006), entrain, and organize faster ones (Monto et al., 2008; Fingelkurts and Fingelkurts, 2017a), providing support for our perspective on this layer being the most basal The timing of localized events across distinct neural assemblies can be coordinated by these slow oscillations (Sirota and Buzsáki, 2005). In our view, these slow oscillations, due in part to being most efficient at long-range communication (Hyafil et al., 2015) by affecting larger populations of neurons compared to fast oscillations (Sirota and Buzsáki, 2005), provide the unifying mechanisms for global coherence needed in a metastable mind. This is because they coordinate distant self-organized neural assemblies (Sirota and Buzsáki, 2005), allowing them to synchronize and form higher-level abstract operational modules (as described by OA). This would allow individual assemblies formed by synchronization *via* fast oscillations (Singer, 1993; Harris et al., 2003) to be a part of a greater operation. Thus, this layer may be the backbone of the integrated experience, uniting the vastly differentiated activity of the brain functionally and spatially.

This base layer consists of ancient and more modern developments that produce and coordinate slow oscillations including cardiorespiratory activity, DMN activity, thalamocortical activity, and other resting-state networks. In addition, this base layer is a fundamental property of individual neurons and neural circuits as spontaneous, baseline activity is a continuous phenomenon that varies with cortical state (Sachdev et al., 2015). The importance of this basal layer is highlighted by the fact it is responsible for the majority of energy usage by the brain (Fox et al., 2005; Raichle and Snyder, 2007). Traditionally considered noise (Chang and Glover, 2009), respiration and cardio activity may entrain or influence these slow oscillations (Fingelkurts and Fingelkurts, 2017a) aiding in long-range communication needed for global coherence (Tong et al., 2013; Tort et al., 2018). Thus, cardiorespiratory oscillations may be included as a component of this layer, and may even support it.

Evidence is mounting on the dramatic effects respiration can have on neural oscillations. As suggested, they may be a part of this lowest layer in providing an oscillatory scaffold for faster oscillations to form. Recent research strongly suggests that the respiratory rhythm may act as a unifier, global coordinator, modulator, and tuner of cortical and subcortical firing and temporal dynamics (Heck et al., 2017; Karalis and Sirota, 2018; Zaccaro et al., 2018). The respiratory rhythm can even dominate local field potentials during sleep (Karalis and Sirota, 2018). This effect largely occurs during nasal breathing *via* the olfactory bulb as projections from it are rhythmically coupled to respiration (Phillips et al., 2012). This olfactory activity has been shown to cause respiration-locked delta oscillations and modulations of higher frequencies in non-olfactory cortices such as the somatosensory cortex (Ito et al., 2014). From the sensory cortex, the respiration-locked activity propagates to other cortical sites that do not receive direct respiratory sensory inputs (Heck et al., 2017). The respiratory sensory inputs into the olfactory bulb drive neurons to fire in the rhythm of the breathing frequency (Heck et al., 2017). Uncertainty, however, lingers when attempting to describe the increase in gamma power that is in phase with the respiratory rhythm (Ito et al., 2014). We suggest that understanding the nature of how fast oscillations entrain upon slower ones will reveal the nature of this phenomenon.

Slower oscillations provide a greater wide-spread resonance in contrast to the distinctly localized effects of faster oscillations (Nunez, 1995). This makes them more suited to act as an organizing and unifying influence. Cross-frequency coupling of faster oscillations with the slow ones is suggested to couple active neural assemblies as described (Buzsáki and Wang, 2012). This global cohering effect is revealed in those with a severed corpus callosum in which the slow oscillation network splits to become independent in each hemisphere (Mohajerani et al., 2010). Slow oscillations produce large, synchronous membrane potential fluctuations in independent neurons throughout brain-wide networks (He et al., 2008) while faster oscillations produce much milder fluctuations in a smaller, more localized extent of cells (Buzsáki et al., 2013). These observations further support our view that the slow oscillations of layer one unify the vast ensembles of neural assemblies into a global framework *via* far-reaching bioelectric fluctuations.

Our perspective on this basal framework may provide insight into “the binding problem,” which ponders how the singular, unified, integrated experience occurs from the numerous and distributed neural activity which contributes to it (Quiton et al., 2010). By entraining diverse oscillations into a coherent whole, these slow oscillations may provide a means of global integration. In line with our theory that the superimposition of the spectral oscillations mirrors the superimposition of phenomenal content, we propose these slow oscillations in part produce an unconscious virtual coordinate matrix upon which all qualia of phenomenal experience are embedded, and thus, these oscillations may be understood as a neural correlate to the phenomenological basis of consciousness (Jerath and Beveridge, 2019b), a 3D virtual space-time matrix (Revonsuo, 2006; Fingelkurts et al., 2010; Jerath et al., 2015a). We experience the world from the perspective centered at the mathematical origin of this space (Trehub, 2007; Blanke and Metzinger, 2009). However, we do not experience the empty coordinate space itself but experience it indirectly as it allows phenomenal contents to come into being (Damasio, 1999; Fingelkurts et al., 2013). In support of survival every-changing environment, this space-time is a direct replication of the physical world allowing a simulation of the external world to be experienced consciously (Siegel, 2006).

**Figure 3:**
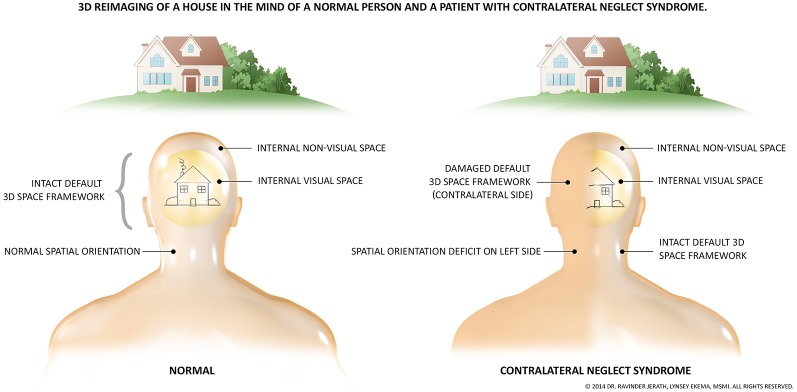
The fundamental presence of an unconscious, 3D, virtual coordinate matrix. The necessity of a virtual, spatial matrix for the existence of the human and likely all animal consciousness is illustrated by the symptoms of contralateral neglect syndrome. This syndrome most often arises from right-hemispheric damage to the right parietal lobe. Due to damage to networks related to spatial processing, one side of one’s perceptual space ceases to consciously exist, illustrated by this figure. As depicted, not only does visual perception disappear, but all perception of that side is removed. These patients are not aware of the missing space, and the side remaining is experienced as the full physical world. Stimuli from the missing side are still processed however, but remain outside of consciousness. The image previously published in Jerath and Crawford (2014); permission provided by Copyright Clearance Center, Inc, Copyright © 2014 Elsevier Inc.

Although our experiences of the external world are internal recreations, we still experience them as externalized (Metzinger, 2003), and they can thus be considered virtual. This phenomenological basis of consciousness is illustrated in the syndrome of contralateral neglect (CN; Figure 3). Conscious quale arguably requires spatial structure to exist (Oizumi et al., 2014). This first layer may provide the necessary spatial foundation for such qualia experienced by all to form. The objects present in our experience of the world (a recreation of the world) which have certain qualia are indeed placed within a 3D space, and this 3D space may be understood as the most fundamental cognitive aspect of our interaction with external reality (Fingelkurts et al., 2013). In addition, aspects of the bioelectric architecture of this layer, such as the functional connections among the DMN, may provide the next component in the hierarchy of the phenomenal mind, the self. The “self” as we use it is a function model of an organism (Metzinger, 2003). Phenomenal content of any type must be superimposed within a virtual space as all experiences have structure (Oizumi et al., 2014). A representation of the self-vs.-other may not be at all essential for complex phenomenal contents to exist (experiences of selflessness), however, for basic homeostatic phenomenal drives (such as hunger) and much of human experience to emerge, a self-representation is required to model “what” is being driven to eat “what.” These homeostatic drives make no sense without a distinction between the self and other.

More primitive scientific views on the singular and integrated nature of consciousness have asserted that information in the brain is a feed-forward process where information is fed to the highest level of the brain where it is experienced (Herzog and Clarke, 2014). In a modern view we share, a global, metastable coherence of reentrant activity leads to the generation of integrated experience by binding local activity with global synchrony (Edelman et al., 2011). The slowest infra-slow frequencies of approximately 0.1 Hz have the most dramatic entraining effects (Sirota et al., 2003) and we suggest they are most responsible for global binding mechanisms. These slowest frequencies are thus not conscious but however provide a physiological and phenomenological basis for conscious contents. By providing a bioelectric foundation for faster oscillations to be “built” upon, this layer of slow oscillations may globally unify faster oscillations scattered throughout the cortex into a greater metastable whole. This layer in turn, according to the identification of isomorphism among the superimposing of the components of these layers, plays the phenomenological role of a virtual, spatial foundation for the contents of consciousness to be “placed” in. Thus, mirroring the biological component, the phenomenal component of this layer provides a unifying structure needed to combine distinct objects and qualia into the integrated experience.

### Layer Two—Primordial Emotions

Layer two provides a fundamental cognitive basis which is survival-focused and potentially a homeostatic means for the brain to interface with the body. This layer determines the general mode of processing for faster frequency activity, constraining behavior and higher thought with basic needs (Knyazev, 2012). It is suggested that Delta oscillations arise from thalamocortical and corticocortical interactions while the infra-slow oscillations arise directly within cortical circuits (Steriade and Mccarley, 2005). These ancient oscillations dominant in the brains of lower vertebrates are primarily responsible for basic homeostatic, motivational, and value processes which include primordial emotions (Denton et al., 2009; Knyazev, 2012). The so-termed primordial, primal, or homeostatic emotions are behavior-driving instinctual sensations compelling one to satisfy biological needs by spurring the overwhelming desire to escape danger or achieve gratification (Denton et al., 2009). These include hunger/thirst for food and air, mating drive, fatigue, and drive to avoid pain and involve more ancient (relative to the “classic” emotions) lower brain structures such as the medulla (Denton, 2006). In addition to subconscious primordial emotions, this layer may be responsible for more unconscious homeostatic processes. Measures of Delta activity do indeed correlate with autonomic and metabolic activity, suggesting a role in synchronizing the brain with the autonomic system (Knyazev, 2012).

The contents of the phenomenal mind consist of more than objects in a virtual 3D space, but also of thoughts, emotions, pain and pleasure. These more abstract experiences, however, are placed within the 3D space as components of “the self” which is the phenomenal and cognitive center of this space. Primordial emotions, as well as advanced emotions, are elicited when something salient happens to an animal. If something has direct relevance to needs, goals, values, or well-being, it will likely stimulate some emotion (Ellsworth and Scherer, 2003). Emotions constrain an animal’s cognitive activities to deal with important events and may produce strong motivational forces (Frijda, 2007). They engage the entire person, cohering and even synchronizing the many subsystems involved in emotion including a tuning of the autonomic nervous system (Scherer, 2005). *Via* the ability of this layer of Delta oscillations to set the general mode of processing in part *via* primordial emotions (Knyazev, 2012), it may provide a means to explain neurophysiologically the phenomenology of primordial emotions. This phenomenology includes basic feelings of pleasure and pain, urgency, cravings, drives, readiness, and inhibition which direct our unconscious and subconscious lives (Scherer, 2005). The unconscious and subconscious mind drastically overshadow the conscious mind in its influence on our actions and experiences (Eagleman, 2011). The ability of lower frequency activity to dictate the activity of higher frequencies provides support for our connection in our layered model between this layer and basic, subconscious/unconscious motivational drives.

Delta oscillations may represent a primitive oscillatory mode with very important motivational roles in primary processes in higher mammals. These oscillations may also provide a (un/sub)conscious cognitive function to screen internal and external stimuli for salient motivational cues that indicate a possible threat or reward (Basar, 1999). While these oscillations have often been suggested to be unconscious (Blumenfeld, 2005), we believe their role in primordial emotions indicates they can have subconscious aspects. This is supported by studies revealing subconscious, threshold perception of stimuli can be attributed to Delta activity in sensory cortices (Parnefjord and Basar, 1999). The basic motivational component of Delta activity is demonstrated in its increase during states of hunger (Knyazev, 2007), sexual arousal (Heath, 1972), sleep craving (Lal and Craig, 2005), reward craving (Reid et al., 2005), and instinctual, impulsive defensive behavior (Knott and Lapierre, 1988; Knyazev, 2012). In addition to the thalamus, waking Delta oscillations are observed to originate in reward and salience circuitry of the brain (Grace, 1995; Gray, 1999). The advanced cognition and emotions of the upper layers are inseparably linked with the survival/motivational aspects (Knyazev, 2012) of this lower layer. We thus assert Delta oscillations play a subconscious cognitive role in constraining the emotional and cognitive activities of the upper layers to focus on biologically salient aspects of the environment. We interpret this research as support for our view that this layer is the most evolutionary old layer that gave rise to fundamental subconsciousness.

The phenomenal aspect of the foundation of this layer, considering the role of the biological oscillations, consists of basic homeostatic drives functionally founded in the most basic division of self-vs-other. Emotional and primordial sensations are thought to have arisen on the back of a more general capacity for basic self-awareness (Purves et al., 2018). The self-representation and cognitive construct of the self in total may arise from temporally related self-representations increasing in complexity from the simple presentations of the body to complex representations of the mind (Craig, 2003). The virtual foundation of the phenomenal and likely cognitive self is a center of origin of the 3D virtual space described by which this dual representation (self-vs-other) is superimposed (Revonsuo, 2006; Blanke and Metzinger, 2009). While the complex sense of self and how this self we experience is related to the underlying 3D virtual space has been associated with neocortical systems such as the DMN (which does change during primordial emotions; Fingelkurts and Fingelkurts, 2017b), we suggest a more basic and less conscious cognitive and sub-phenomenal self-representation may arise from more primitive representations in the brainstem. These simpler representations may account for a phenomenal self and thus allow for primordial emotions in animals (of which this layer is dominant) which do not have a neocortex.

Regardless of the anatomical origin, the most basic self would allow a representational basis required for the homeostatic and motivational sensations such as hunger to be realized. These phenomenal drives of this layer are thus entrained upon basic representations of the self as well as upon what is modeled as the external world. The external objects and internal sensations related to the self are themselves entrained upon the homeostatic phenomenon of this layer giving them survival meaning. This would create a dynamic between the model of the self and of the external world that allows for survival-relevant behavior. It is beyond the scope of this article as to what role oscillations have in the development of this most basic cognitive and phenomenal self. According to our interpretation, all of the remaining layers are entrained upon this one and thus are still ultimately constrained around basic survival drives. According to theories of embodiment, higher mental functions are shaped by the way we are in the most fundamental aspect of the self, embodied and by how our bodies interact with the world (Wilson, 2002). This is revealed by the fact that simple representations of the brainstem can alter higher-order cognition such as emotional phenomenon (Venkatraman et al., 2017) attributed to the next layer we describe.

### Layer Three—Advanced Emotions and Cognitive Fundamentals

Emotions are key aspects of phenomenal experience (Damasio, 1999). The third layer we will now explore is used by the still ancient but relatively advanced emotional circuits which have important survival and social functions necessary for effective interaction with the social and physical environment. Emotional related activity from the limbic system *via* dense connectivity strongly influences neocortical activity during emotional processing, greatly affecting how we sense and think about the world. This interaction between the limbic system and neocortex is thought to be responsible for the phenomenal feelings of emotional states which can have a powerful influence on cortical faculties responsible for logic-based cognition (Purves et al., 2018). The diffuse, ascending modulatory pathways of the limbic system play a strong role in the selection of activity and activity patterns (Edelman et al., 2011) in the top layer we describe next. Indeed, this layer of Theta and Alpha oscillations used by the limbic system dominates the EEG of the waking mammal (Klimesch, 1999; Clayton et al., 2018). This may correlate to why mammalian thinking and behavior is much more influenced by emotion than logic (Medina, 2008; Luo and Yu, 2015). In addition, the strong tie between this layer used for emotions and the top cognitive layer may correlate to the strong interaction between emotion and common logic (Jung et al., 2014). There is a strong tie between the base layers and this largely emotional layer as well. As respiratory activity may be a key modulator and the entrainer of the base layers (Zelano et al., 2016), the interaction between these layers may explain the close tie between respiratory activity and emotional states (Masaoka and Homma, 2005; Homma and Masaoka, 2008). In addition to higher-order emotions, activities implementing this layer of oscillations may also provide basic functionality for the most fundamental cognitive operations such as attention and working memory.

Theta band synchronization plays a key role in emotional processing (Knyazev, 2007) including mediating the detection, integration, and evaluation of emotionally salient stimuli (Symons et al., 2016). Theta activity is most associated with limbic structures, and electrical stimulation of the limbic system evokes distinct Theta activity (Gray, 1982). This band is also commonly associated with fundamental (un/sub)conscious cognitive activity such as the integration of new information with internal representations and other memory functions (Klimesch, 1999). While consciousness may seem to be continuous in time, it may actually consist of discreet “moments” that last a certain small time frame. It has been suggested that Theta activity may underlie the “frame-rate” of conscious experience by setting the time scale required for the integration of the vast, spatially distributed local computations of the higher layers; the cycle time of Theta activity does indeed match the biophysically measured duration of subjective presence (“frame-rate”; Singer, 2011). If this is accurate, then the higher frequency activities superimposed on the Theta activity may mirror how phenomenal contents are superimposed into time.

The direct and indirect (*via* the thalamus) connections between the limbic system and an array of neocortical areas (corticolimbic circuits) are key to higher-order processing of emotions (Purves et al., 2012; Hultman et al., 2016). Studies have repeatedly shown the ability of emotional processing to influence cognitive processing (Ray and Zald, 2012). Transient coupling of rhythms between distinct and distant structures can guide bidirectional information transfer between such structures (Sirota and Buzsáki, 2005). In the waking mammal, the highly prominent Theta oscillations in the hippocampus functionally define the limbic system as the entire limbic anatomy are modulated by such oscillations (Buzsáki, 2002). Tight synchronization in the Theta band among corticolimbic circuits such as between the hippocampus and prefrontal cortex are fundamental not only in emotional processing (Jin and Maren, 2015) but in basic cognitive functions such as spatial learning and memory processing (Colgin, 2011; Gordon, 2011). The paleocortex is also tightly coupled to this Theta activity of the hippocampus, and the paleocortex is unified with the neocortex *via* oscillations (Sirota and Buzsáki, 2005) which may provide an additional means for emotion to modulate the cortex.

As a part of our model, we describe subconscious to conscious Alpha oscillations as the frequencies which helps create unique “empty” sensory frameworks which bring the unconscious virtual space into greater modes of awareness. Thus, alpha activity often represents these sensory frameworks entering spatial awareness. This hypothesis is supported by the fact that these oscillations are known to influence perception (Iemi et al., 2017) and determine if a near-threshold stimulus enters awareness or not (Mathewson et al., 2011; Thut et al., 2012), and are revealed in sensory cortical areas when one closes their eyes, removes, or otherwise eliminates incoming stimuli to a sensory modality. Similar to how the underlying 3D coordinate matrix created by the lower layers is an unconscious phenomenological foundation of all consciousness, the phenomenologically unique sensory modalities which are integrated within this matrix have structurally unique subconscious oscillatory networks which produce the virtual framework of specific sensory consciousnesses such as vision. Thus, in line with the main assertion of this article, we suggest that the nature of how certain operations of this layer which may form the cognitive and phenomenal frameworks for the various sensory modalities are entrained upon the lower layers we have suggested produces and consists of the underlying 3D space. Furthermore, vivid qualia from the top layer we next describe become embedded in the sensory frameworks which use this layer which are themselves embedded in the virtual 3D matrix of the base layer. This layer of oscillations, primarily in Alpha frequency, form the foundation for bringing the sub-phenomenal 3D space into consciousness; spatial attention. These oscillations are essential to the fundamental awareness of any sensation or space and are, according to our assertion, superimposed upon the base layer of oscillations creating the virtual space just as the phenomenal sensory experiences are superimposed upon it. According to our model and empirical observations, when these oscillations are lacking in underling higher frequency activity which would normally be integrated into consciousness, the consciousness of such activity is eliminated (Fingelkurts et al., 2012). This is illustrated in contralateral neglect syndrome (CN) which has traditionally been thought to arise from anatomically specific damage to certain processing centers such as the parietal lobe, however, due to the wide range of sites that when damaged lead to CN, structural damage to specific areas is insufficient to fully describe the mechanisms leading to the neglect (Corbetta and Shulman, 2011; Corbetta, 2012). Disruption of the spatial attention networks in the Alpha band by right-hemispheric damage has been shown to result in CN (Sasaki et al., 2013). Thus, we suggest this layer brings the unconscious, virtual, 3D coordinate matrix we have described into consciousness.

In addition to the isomorphism between the superimposition of these Theta to Alpha oscillations onto lower frequency ones and of the sensory modalities onto the underlying 3D space, there also exists an isomorphism between emotional aspects of this layer and the underlying layers. There are likely more complex dynamics to this superimposition isomorphism as well which are beyond the scope of this article. Emotions are thought to be mediated from certain dedicated neural circuits (LeDoux, 2012), or as we suggest, bioelectric processes of neural assemblies. A cognitive purpose of emotion can be thought to be to, in acting like a working memory, sustain the various neural activity which performs relevant to states of the world which require certain mental and behavioral reactions (Purves et al., 2018). The classic emotions promote survival on a more abstract level than the primordial emotions described in the previous section, adding complexity to them (Montag and Panksepp, 2017).

The diversification of emotional experience (beyond primordial emotions) throughout evolution may have developed to not only to serve communicative functions but facilitate a proper mental and behavioral response to solving a diverse range of adaptive problem domains that influence the chance of reproductive success (Al-Shawaf et al., 2016). Emotions are in a sense phylogenetic neural algorithms that facilitate decision making and thus behavior by providing approximate solutions to potentially complex survival-relevant situations in which an optimal behavior is not neurocomputationally tractable (Bach and Dayan, 2017). Most importantly to our hypothesis, emotions have been described as “superordinate mechanisms,” being higher in a hierarchy in the sense that they appropriately (according to an organization dictated by reproductive fitness) coordinate assemblies of other cognitive operations such as attention and perception (Tooby and Cosmides, 2008). We thus suggest that these modern emotions are phenomenally, physiologically and cognitively superimposed upon more primitive drives such as self-preservation, the drive to avoid pain, and the drive to achieve pleasure. While emotions can coordinate the complex cognition and perception present due to the fourth layer, they are themselves coordinated by the survival-focused activity of the second layer. For instance, emotions such as happiness and sadness may be cognitively and phenomenally entrained upon the primordial sensations of safety or satiation.

### Layer Four—Higher Cognition and Vivid Sensory Consciousness

The upper layer of our model consists of more localized, infrequent, higher frequency Beta and Gamma oscillations (>12.5 Hz) and may be responsible for higher-order consciousness necessitated by the lower layers responsible for primitive consciousness. We suggest this layer is responsible for sensory consciousness, sensory qualia, and higher cognitive functions which we suggest are superimposed cognitively, phenomenally, and physiologically upon the respective components of the lower layers. For instance, sensory qualia are superimposed upon the sensory frameworks of which they correspond. Certain cognitive capabilities are coordinated by emotional activity or “affect programs.” In essence, these higher levels of cognition and sensory experience are dependent on the lower layers we have described to be part of a global metastable continuum.

There is still debate among neuroscientists as to whether Gamma oscillations ( >30 Hz) play a functional role in consciousness and cognition, from being completely non-functional to being a neural correlate of consciousness (Muthukumaraswamy, 2019). Due to this lack of scientific consensus, we will suggest some hypothetical functions of Gamma in relation to being a part of this layer, while still recognizing that the evidence for the significance of these waves is lacking. Our interpretation of this debate is that while there is significant support for the idea that Gamma oscillations are a by-product or noise, we assert that when superimposed upon a full bioelectric layered structure described thus far, they indeed may have an important function. Opposition to the relationship between Gamma oscillations and the cognitive and phenomenal mind points to research suggesting that in part, the readings of these oscillations may arise as artifacts from the dynamics of miniature muscular saccades (Whitham et al., 2008; Yuval-Greenberg et al., 2008)The frequency of muscular activity overlaps with the Gamma band, and so traditionally, the Gamma was filtered out of the EEG (Muthukumaraswamy, 2019). While a vast majority of the Gamma activity can be ascribed to muscular activity, some still remain upon complete muscular paralysis of the body (~1% of non-paralyzed power; Whitham et al., 2007).

There are many other aspects of this debate. For instance, the dynamics of the Gamma band have been asserted to be an insignificant byproduct of power changes in the lower frequency bands (Pulvermüller et al., 1995). It can be argued that while there are many studies showing powerful and statistically significant associations between the Gamma band and cognitive functions, it is argued that these cannot currently be meaningfully interpreted (Fingelkurts and Fingelkurts, 2010b) given the said limitations and those of spatial filtering (Robinson et al., 2001), noise, harmonics of lower frequency activity (Freeman, 2003), lack of significant contribution to the full spectral power (Thatcher, 2001), and lack of rhythmic quality (Bullock et al., 2003). While we cannot currently determine the nature of Gamma oscillations in relation to cognition and the phenomenal mind, we suggest that by analyzing them in relation to how they are superimposed upon other oscillatory structures to form a greater operation, we may begin to understand their role and why they are simultaneously proposed to be associated with complex cognitive processes and even consciousness while still being realized in invertebrates (in response to simple stimuli; Schütt and Başar, 1992), anesthetized states (Steriade et al., 1996), and apparent gestalt binding responses to meaningless stimuli (Trujillo et al., 2005).

Sensation does not require oscillations or even a nervous system to occur as seen in the sea sponge (Leys, 2015). In our opinion, however, sensory consciousness does require a layered and organized oscillatory structure to appear, and the nature in which phenomenal sensory contents are superimposed on the objects and scenes present in consciousness reflects the nature in which oscillations related to sensory qualia are superimposed on oscillations related to the sensory frameworks and objects and scenes we experience. Fast oscillations have relatively localized effects, and so in addition to the slow oscillations they “ride” upon, they are coordinated by the reentrant activity of the corticothalamic network. Phase synchrony of Beta and Gamma waves is enhanced for stimuli that arise into consciousness (Meador et al., 2002; Palva et al., 2005), particularly when this synchrony reaches a global level (Melloni et al., 2007). We suggest this is due to this activity entraining upon the lower layers. The fact that mammalian sensory responses to external stimuli during sleep are evident in Delta activity (Basar, 1999) while during wakefulness evident in Gamma waves (Welle and Contreras, 2016) in part supports our view that this third layer of oscillations is responsible for sensory consciousness. Our view on Beta and Gamma waves as the correlate of higher cognition and sensory perception is further substantiated (although this evidence is correlational) by a myriad of works potentially implicating this activity in sensory representation (Eckhorn et al., 1993), integration (Engel and Singer, 2001; Schoffelen et al., 2011), acquisition (Ribary, 2005), along with planning, information processing (Ribary et al., 1991), memory, top-down modulation (Stookey et al., 1941), attention (Herrmann and Knight, 2001), perception, and consciousness (Abhang et al., 2016).

The EEG and EGoG (Electrocorticography) literature in general support or view that lower frequency activity represents more basic and widespread activity while higher frequency activity is more localized and information-rich (Orpwood, 2017). While Beta activity correlates with behavioral choices (Donner et al., 2009; Haegens et al., 2011) and is suggested to be a maintainer of sensorimotor and cognitive state (Engel and Fries, 2010), we assert the importance of how this Beta (and possibly Gamma) activity is entrained upon the lower frequency activity in determining the cognitive, sensorimotor, and behavioral outcomes. The higher frequency activity of this layer has also been associated with metacognitive processes and insight problem solving (Rosen and Reiner, 2017). This supports our view that higher frequency activity is related to more complex and abstract cognitive and phenomenal content.

We have included the body into this layer by proposing a novel concept of top-down effect which is also a part of the sensory activities of this top layer. This novel view extends the current realization that strongly influential top-down influence may extend to the dorsal horn of the spinal cord *via* descending projections (Purves et al., 2018). In the view we espouse, descending projections not only extend to the sensory receptors themselves *via* membrane potential oscillations, but both the ascending and descending pathways are synchronized into one unified resonance (Jerath et al., 2016). *Via* the functional connectivity of electromagnetic synchronization, these oscillations essentially bring the processing capabilities of the cortex to the sensory receptors for efficient and task-relevant interpretation of the external world (Jerath and Beveridge, 2019a). The membrane potential Gamma oscillations along the afferent sensory axons would allow the sensory receptors to be primed to attend to, expect, or ignore certain information. In addition, by maintaining sensory representations at the site of the sensory receptors, some of the need for complex mental representations can be alleviated. Future research should investigate the existence and role of membrane potential oscillations along peripheral sensory afferents.

## States

**Figure 4:**
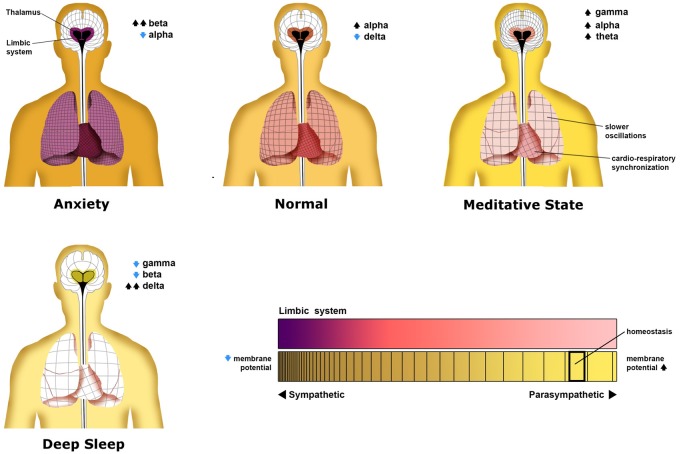
Patterns neural oscillations in various neurological states. This figure illustrates the distinct oscillatory patterns that commonly occur in the average person. Each panel shows not only oscillations in the central nervous system but non-neural oscillations of the heart and lungs. As the thalamus is proposed as a central hub for thalamocortical oscillations in the central nervous system, it has been placed as the center of the neural grids shown in each person. The density of each grid represents the average frequency of oscillations across the cortex, lungs, and heart, with higher densities representing higher frequencies. Surrounding the thalamus is a colored representation of the level of limbic system constraint on cortical activity of which deep purple represents the most limbic constraint. In the deep sleep state, a limbic constraint is eliminated as there is no behavioral cognitive activity. This is represented by an olive color. Although not discussed, global membrane potential and autonomic tonic are represented by the color of the person’s skin. These states are discussed in detail in the text. This figure is an original image done by Michael Jensen.

Different states of mind have been identified with predictable global oscillatory patterns (Saggar et al., 2012; Figure 4). In part from the vast body of literature correlating electrophysiological activity to mental states, the global state of one’s bioelectric network is suggested to be isomorphic to the state of one’s consciousness (Fingelkurts et al., 2010, 2013). By describing these states as varying superimpositions of phenomenal contents along with mirrored superimposed oscillatory layers, we may further develop understanding of the nature of mental states in relation to their isomorphic bioelectric neurophysiology. The implications for understanding the physiologic nature of positive and negative mental states may include the advancement of treatment for mental conditions arising from aberrant neural systems and improved techniques for promoting general well-being.

The states we describe and the research we review in regards to providing support for the superimposition model may help reveal how the integrated conscious experience arises from not from synchrony alone, but from a metastable hierarchy of synchrony in which local circuits are harmonized with a global network. This is revealed by the unconscious, hypersynchrony of deep sleep and epileptic states in which the entropy of brain activity is low (Guevara Erra et al., 2017) and the oscillations are low/high in frequency respectively (Kostopoulos, 2001). On the other hand, a brain with a complete lack of large-scale synchrony altogether will surely lack conscious experience.

### Sleep

Sleep has been divided into several stages by sleep scientists. These are further differentiated by two main types of sleep, REM sleep, and the slow-wave NREM sleep. Slow-wave sleep is characterized by the emergence of slow membrane potential oscillations throughout the brain as well as macroscopic Delta activity (Niedermeyer and Lopes Da Silva, 2005). REM Sleep is characterized by EEG activity very similar to activity in the waking state (Roohi-Azizi et al., 2017). The transition from wakefulness into sleep is characterized by an increase in the theta rhythm, reflecting a slowing of overall frequency (Merica and Fortuneb, 2004). Individual neurons throughout the entire brain exhibit infra-slow membrane potential oscillations of the first layer during slow-wave sleep (Steriade and Mccarley, 2005). From our standpoint on sleep, one role of these slow, global membrane potential oscillations during sleep is to restore imbalanced membrane potential excitability homeostasis *via* slow hyperpolarization (Jerath et al., 2014b) as extended waking time leads to over-excitability of the individual neuron (Winters et al., 2011; Yan et al., 2011).

During slow-wave sleep, sensory consciousness is not present. The absence of this top layer and the second layer in sleep leaves the lower level bare. This may allow the ancient, fundamental slow oscillations of the first layer to rise in an activity which may be why they dominate the sleep EEG (De Vera et al., 1994). Returning to a prototypal stage of brain activity may be necessary to reconcile day-time experiences into the fundamental structural coding of the brain (Knyazev, 2012) and more specifically the more primitive brainstem and cortical regions such as the DMN which are responsible for basic biological and self-referential processes. Insomnia has been associated with alterations in fundamental EEG activity related to sleep. This may reflect the failure of the lower layer to emerge due to the failure of the higher layers to shut down. Insomnia patients often exhibit an abnormal presence of Beta activity during NREM sleep and during sleep onset (Merica et al., 1998; Nofzinger et al., 1999), which is negatively correlated to the quality of sleep. This activity may correlate to the phenomenology of insomniacs who experience hypervigilant or excessively ruminating states when trying to fall asleep (Perlis et al., 2001). Without a bioelectric structure of superimposed layers active during deep sleep, we suggest the remaining, bare base layer provides only a basic subconscious state. Consciousness has often been thought to disappear during deep, dreamless sleep; however, some researchers have argued that good empirical and theoretical reasoning exists for the presence of some sort of dreamless sleep-experience (Windt et al., 2016).

### Chronic Distress

The emotionally constraining states of distress of which we also include depression and anxiety are experienced by a growing number of people and thought to be more widespread and profound in modern times (Schneiderman et al., 2005). Distress is differentiated from its performance improving counterpart eustress, which is experienced during desirable events in one’s life (Selye, 1974) such as riding a roller coaster or playing sports. Distress produces a negative emotional reaction which includes fear and a sense of loss of control (Hamid et al., 2010). Chronic distress can have serious deleterious effects on one’s quality of life and health (Giannakakis et al., 2015; García-Martínez et al., 2017). While many studies have been conducted looking to the EEG of the distressed brain, there are inconsistencies in the results likely due to subject variability (Tran et al., 2007). Although frequency-based analysis may not be the best approach to studying the distressed brain with EEG (Valenza et al., 2012), many studies have demonstrated an increase in Beta activity and decrease in Alpha activity during distress (Reisman, 1997; Hamid et al., 2010) in certain brain areas such as the right frontal lobe (Macaulay and Edmonds, 2004; Fingelkurts et al., 2006), and more recently increases in relative Gamma activity has also been tied to distress in these similar frontal brain areas (Minguillon et al., 2016). Stress, anxiety, paranoia, and an inability to relax are associated with over-dominance of high-frequency range activity (Abhang et al., 2016). Chronically stressed persons have been shown to exhibit steady beta reactions to unpleasant stimuli while non-stressed persons show decreasing beta activity with exposure time demonstrating stress-resistance abilities (Hayashi et al., 2009).

Decreases in Alpha activity along with increases in Beta activity also occur during an intense mental activity such as when answering exam questions (Niemiec and Lithgow, 2006). Our interpretation of findings correlating increased Beta and decreased Alpha (Chandra et al., 2017) activity in the chronically distressed can be understood in a similar fashion to the autonomic nervous system. Unexpected sudden stimuli may provoke short-term stress, stimulating a response to protect the animal (Steriade et al., 1993b). Just as Beta power is correlated with an intense mental activity that may occur during this normally short-term stress, the sympathetic nervous system is activated during similar times of intense short-term stress. However, this intense arousal activity was evolutionarily purposed for fleeting “flight or fight” moments, not continuous arousal (Nesse et al., 2016). Something about our frenetic modern way of life whether it be our stressful jobs, sleep deprivation, or our tendencies to commit ourselves to goals we cannot achieve and responsibilities we cannot fulfill is causing our evolutionarily purposed, short-term stress mediating sympathetic system to be activated continuously, most often with the lack of physical exercise (Wang et al., 2010; Nesse et al., 2016; García-Martínez et al., 2017).

Chronic sympathetic activity can have many degrading effects on physical and mental health due to this evolutionary mismatch between prehistoric and modern ways of life (Fisher et al., 2009). In this autonomic system comparison, chronic activation of the arousing upper-layer activity meant for relatively short, but intense moments may occur due to its tendency to promote itself in our modern society. It may promote itself because high levels of distress can produce a mental blockage that may make it almost impossible to properly solve problems, leading to more distress (García-Martínez et al., 2017). We suggest, due to the phenomenological similarities of cognition (not arousal) between the chronically distressed state and during sleep deprivation, that just as in sleep deprivation, increased neuronal excitability and activity levels may correlate with this mental blockage (Yan et al., 2011). The increased activity of the upper cognitive layer during distress may be localized to areas of the brain purposed for producing negative experiences (Roohi-Azizi et al., 2017) which would motivate one to remove themselves’ from some situation, thus making one highly aware and cognitive of these negative thoughts and feelings.

During transient times of stress, this upper layer activity may promote greater awareness over the task-demand; however, chronic distress likely diminishes cognitive flexibility and awareness constraining mental activity to lock into certain over-active, impulsive processing modes associated with anxiety and depression. The phenomenology of the alert states of distress and long-term meditation are drastically different. In the distressed state, the heightened activity of one’s mind may feel enforced by the brain and one may feel as if it is taking a significant amount of effort not only engage the task but to quiet the mind. In the meditative state however, the increased cognitive activity is effortless and one is free to think free of emotional constraints. We also suggest that wave properties (power, phase-locking, symmetry, etc.) of the higher-frequency activity observed in the contrasting states of distress and higher awareness achieved through meditation are fundamentally different and this notion should be researched further. One likely difference is an asymmetry of oscillatory activity in the two hemispheres of the brain in the distressed state, with symmetrical sites of the cortex showing large differences in the frequency of oscillations present (Deldin and Chiu, 2005).

Distress not only results from changes in the frontal lobe areas we have described but consists of coordinated activity between these areas and the limbic system (Karalis et al., 2016). Synchrony between these distal neural areas may provide a means for the limbic system to constrain the activity of the cortex as the low-frequency oscillations that mediate this long-range corticolimbic synchrony can organize the activity of local cortical neural assemblies (Karalis et al., 2016). Individuals with high-anxiety show increased connectivity between the relevant limbic and cortical areas (Vytal et al., 2014). Thus while the distressed state may show high-frequency activity that would normally indicate increased awareness and cognitive activity, we propose as a part of our model that this activity initially represents normal, important, emotionally constrained cognitive function, however it becomes dysfunctional long-term possibly due to long-lasting, functionally and anatomically neurotoxic effects of excessive hormones released by the sympathetic nervous system (adrenaline, cortisol; Chetty et al., 2014), leading to a lack of cognitive functioning despite mental stimulation, and an over-awareness of negative feelings. Research has shown that respiration pattern may globally entrain brain rhythms allow for the voluntary control of traditionally involuntary mental and bodily processes, and thus by modulating the body, one may modulate the mind. Due to the extensive connections among the heart, lungs, and autonomic nervous system with the limbic system, we assert that by purposely modulating cardiorespiratory activity *via* deep breathing, one may be able to attenuate the over-arousing and unpleasant effects of the sympathetic and limbic system on the cortex and thus decrease distress (Jerath and Barnes, 2009; Jerath et al., 2014a, 2015b).

Just as the lower frequency activity constrains the higher frequency activity based on the nature of how the higher frequency activity is superimposed upon the lower frequency, the higher-order phenomenal and cognitive aspects of distress are constrained by the lower-order aspects based on how they are superimposed upon the lower-order aspects. As mentioned, the distressed state has ties to the autonomic response purposed for short-term survival reactions. We suggest in the distressed state, the activated Beta activity and the cognitive and phenomenal capacities that emerge from it are bound to the survival circuits of the lower layer. The cognitive processes that occur in this state may be constrained by “primordial emotions” which in this case may be a vicious disposition to “fight-or-flight” one’s way out of some situation perceived as dangerous. In addition, phenomenal sensations that enter consciousness can be strongly concentrated and in a sense superimposed upon emotional and other attentional drives relevant to some external stressor. The lower layers we have described thus form attention driving bioelectric circuits which determine what externalized phenomenal representations while be realized.

### Waking Awareness

The waking state may be divided into at least two states both dominated by Alpha and Theta activity;, active wakefulness and the quiet wakefulness state achieved during daydreaming, sleep deprivation, and mental rest. These states are in part differentiated by the presence of Delta oscillations in membrane potential (Sachdev et al., 2015). Alpha oscillations often dominate during this quiet state, and although Delta activity has been associated exclusively to sleep states (Gaspard et al., 2013), it may also dominate around 10% of the total activity during the quiet waking state in which cortical neurons may transiently go “offline” as if in sleep (Vyazovskiy et al., 2011; Sachdev et al., 2015). While this Delta activity of EEG and membrane potential oscillations are not commonplace, it may transiently dominate local cortical regions (Crochet and Petersen, 2006; Zagha et al., 2013) allowing distinct neural assemblies to become more globally synchronized, supporting our view that the low-frequency membrane potential and macroscopic oscillations provide an underlying widespread synchronizing function.

The active awake state is much more metastable than the quiet state, consisting of locally desynchronized circuits which are coherent at the large-scale vs. the lower entropy synchronization of the quiet state. The fluctuations in membrane potential of individual neurons and in the EEG in this state are dominated by higher frequency oscillations such as Alpha and Theta (Vyazovskiy et al., 2011). Even in this active awake state, Delta activity may dominate in a small fraction of recording sites. In line without our hypothesis on the lower layers supporting the higher layers, this waking delta has been suggested to facilitate the background activity and operation of awake cortical circuits. Sleep deprivation results in a quiet waking state that is more similar to the sleep state in that the lower layer increases in power while the higher layer activity such as Gamma is significantly decreased (Li et al., 2008). This may reflect the need for the homeostatic functions of the lower layer to emerge after disrupted homeostasis caused by prolonged wakefulness.

### Meditative States

Meditative practice includes techniques that develop long-lasting psychological traits and benefits (Baijal et al., 2011) through focused awareness of the contents of one’s experience such as thoughts, emotions, and sensations (Saggar et al., 2012). Aside from the focused awareness, meditation also involves voluntary relaxation of cognitive activity along with muscular relaxation (Cardoso et al., 2004). Meditative techniques include the culturing of different mental states which are expressed in different EEG patterns (Hinterberger et al., 2014). Extensive meditative practice may provide insight and understanding of one’s own phenomenology and lead to lasting improvements in cognitive functioning and well-being (Wallace and Shapiro, 2006). According to our opinion and theoretical model, the dynamic changes in oscillatory architecture in advanced meditators reveal a higher state of awareness and well-being in which thinking is less constrained by emotional and neurotic impulses and in which consciousness may actually be quantitatively greater. A quantitative aspect of consciousness has been proposed as a part of prominent consciousness models (Oizumi et al., 2014) and may be self-evident in one’s personal phenomenology of transitioning from sleep to wake.

While different meditation types result in distinct bioelectric patterns, experienced meditators, in general, show global increases in oscillatory activity (Lee et al., 2018). Despite the growing neuroimaging research with EEG into meditation (Braboszcz et al., 2010), no consensus has yet been drawn on the distinct effects on the EEG by meditation (Braboszcz et al., 2017). Alpha and Theta waves have been shown to increase in power and synchrony with the amount of meditation training (Dentico et al., 2016; Lee et al., 2018). Many recent findings on brain waves in long-term meditators of many types have found that they often have great increases in the amount of high-frequency Gamma activity (>60 Hz) in various brain areas such as the cingulate, parietal, occipital, and somatosensory cortices which correlates with the meditative experiences (Lutz et al., 2004; Berkovich-Ohana et al., 2012; Hauswald et al., 2015; Braboszcz et al., 2017). Although as mentioned, much of the observed Gamma power can be attributed to muscular artifacts. However, when controlling for these artifacts using independent component analysis, increases in Gamma activity have been asserted to remain (Braboszcz et al., 2017).

While there are varying results in EEG patterns associated with different forms of meditation (Kaur and Singh, 2015), similar meditation styles largely have shown similar results. Similar to how delta oscillations during sleep may provide a restorative function, increased delta oscillations during meditation may help promote an enhanced state of wakefulness when meditation is complete (Lee et al., 2018), and may represent the inhibition of cognitive engagement during the practice (Cahn et al., 2010). The increased Theta Activation in neural areas of meditators responsible for autonomic control and internalized attention (Braboszcz et al., 2017) may reflect the means by which mediation promotes a parasympathetic dominant state (Lee et al., 2018). Reductions in beta bands have also been reported which may correspond to changes in self-hood (Hinterberger et al., 2014). Theta activity normally increases as a general result regardless of mediation type or experience level and may reflect an increased level of relaxation (Jacobs and Friedman, 2004; Cahn and Polich, 2006).

In addition to increases in Alpha and Theta power, the freedom from stress, anxiety, and depression achieved by high-level meditators is reflected in findings in even low-level meditators that meditation leads to a reduction in stress-related amygdala resting-state functional connectivity (Taren et al., 2015). Such literature supports our view on the meditative state being one freed from constraining effects of the limbic system and negative emotional and cognitive activity in the cortex which we have discussed in relation to distress. Thus in this state, emotional and cognitive aspects of the upper layer are less constrained by those of the lower layers which are focused on more primitive survival. In contrast to the distressed state, the cognitive activity and heightened awareness of the meditative state we describe is cultured through many hours of mindfulness and/or other psychological exercises characterizing meditation. This meditative state can in part be characterized by our model as an increase in cognitive clarity and perceptual vividness.

Gamma increases are shown to extend to the dream sleep of advanced meditators as well (Ferrarelli et al., 2013), corresponding with increased vividness and reportability of dreams in advanced meditators (Faber et al., 1978). Our interpretation of this abundant research into upper layer related changes is reflected in our layered model which elucidates a meditative state of consciousness correlated with a dramatic “thickening” of the top layer of our model. Perceived and non-perceived words are both associated with local Gamma activity, however, long-distance Gamma synchrony (facilitated by the lower layers) has been strongly associated with the perceived words (Melloni et al., 2007). The increased long-distance synchrony and dominance of this top layer in meditators may suggest some type of quantitative increase in perceptual vividness and/or consciousness. Similar high-frequency activity in the chronically distressed may reveal a similar state of wakefulness, however, this state is brought about by the hormones and arousal of the short-term, defensive stress stress-response which can be very harmful after long-term exposure, ultimately degrading cognition despite arousal (Minguillon et al., 2016; García-Martínez et al., 2017). In the meditative state, activity of the top layer is still superimposed upon the lower layers, however, it does not appear to be as nearly phenomenally and cognitively constrained by them as in the distressed state. If our hypothesis on the isomorphism of superposition is accurate, future studies should find that the dynamic relationship between the faster oscillations and the slower will differ in some fundamental way.

## Conclusion

In this theoretical article, we have advanced a novel perspective on the hierarchical nature of biological oscillations which identifies an isomorphism among the frequency-based superimposition of neural oscillations and the superimposition of the contents of consciousness. The contents of consciousness have been previously proposed to consist of a superimposition of qualia upon objects and scenes further superimposed upon a 3D virtual coordinate matrix. We expand upon this assertion by including emotions and some aspects of cognition. This superimposition is identified *via* a model of a layered oscillatory architecture we believe may describe in part the architecture of a global bioelectric continuum which has been proposed to realize the emergence of phenomenal consciousness. In this model, the widespread lower layers consisting of lower frequency spectra provide a means of coordination, grouping, and unity for the more local, computational, high-frequency activities of the higher layers. The lower layers thus support, modulate, and entrain the higher layers which may, when synchronized with and superimposed upon a greater metastable framework, produce advanced phenomenal experiences (sensory experience, emotions, metacognitive experiences). We support the assertion that the integrated experience of consciousness is thus achieved when the myriad of distinct and highly-local high-frequency synchronies among neural assemblies are metastably synchronized together at a much more global, abstract, and complex scale. While the hypothesis we have presented is not based on our own empirical research, we believe the significance of our assertions may provide a deeper understanding on the nature of the previously proposed isomorphism between the integrated phenomenal experience and the global bioelectric architecture of the brain. Understanding the full nature of this isomorphism will be key in bridging the explanatory gap between the phenomenal mind and biological mechanisms of the brain and in developing theories on the biological and ultimately physical nature of consciousness.

## Author Contributions

Theory developed by RJ with some writing. CB wrote the majority of the manuscript with theoretical contributions. Images done by MJ.

## Conflict of Interest

The authors declare that the research was conducted in the absence of any commercial or financial relationships that could be construed as a potential conflict of interest.
